# Strength Training with Superimposed Whole Body Vibration Does Not Preferentially Modulate Cortical Plasticity

**DOI:** 10.1100/2012/876328

**Published:** 2012-05-02

**Authors:** Ashleigh T. Weier, Dawson J. Kidgell

**Affiliations:** Centre for Physical Activity and Nutrition Research, School of Exercise and Nutrition Sciences, Deakin University, Melbourne, VIC 3125, Australia

## Abstract

Paired-pulse transcranial magnetic stimulation (TMS) was used to investigate 4 wks of leg strength training with and without whole body vibration (WBV) on corticospinal excitability and short-latency intracortical inhibition (SICI). Participants (*n* = 12) were randomly allocated to either a control or experimental (WBV) group. All participants completed 12 squat training sessions either with (WBV group) or without (control group) exposure to WBV (*f* = 35 Hz, *A* = 2.5 mm). There were significant (*P* < 0.05) increases in squat strength and corticospinal excitability and significant (*P* < 0.05) reductions in SICI for both groups following the 4 wk intervention. There were no differences detected between groups for any dependant variable (*P* > 0.05). It appears that WBV training does not augment the increase in strength or corticospinal excitability induced by strength training alone.

## 1. Introduction

Whole body vibration (WBV) has become a popular method of neuromuscular training due to the recent emergence in the benefits of vibration on neuromuscular performance. These benefits have included improved strength, jump height, power, flexibility, and balance (for reviews see [1, 2]). For this reason, it is believed that strength training with WBV may provide superior training outcomes (i.e., increased strength development) compared to traditional strength training methods alone [3–6]. However, many of the studies that indicated beneficial outcomes were either acute studies or training studies that used direct methods of vibration applied to the muscle belly or tendon as opposed to WBV. Therefore, little is known about the training-related effects of WBV on strength development and neural activation. Several studies have compared squat training with superimposed WBV (*f* = 50 Hz, *A* = low) to squat training alone (i.e., without WBV) [7, 8] and found no significant difference between groups for squat strength following six weeks of training. However, it must be noted that the vibratory stimulus used in these studies was applied prior to and after training sets rather than during the training itself. At this point, there still exists a lack of adequate research investigating the effectiveness of WBV as a training tool to improve strength compared to an equivalent training program in the absence of WBV.

Potential mechanisms for improved neuromuscular performance following exposure to vibration include the tonic vibration reflex, vibration-induced increases in the gravitational load on muscle (modulating muscle stiffness to dampen the vibrations), increased corticospinal excitability, reduced short-latency intracortical inhibition (SICI), and increased intracortical facilitatory processes related to muscle activation (for reviews see [2, 9]). Changes in synaptic activity and modulation of neural transmission such as corticospinal excitability and SICI can be objectively assessed through the use of transcranial magnetic stimulation (TMS). TMS applied over the primary motor cortex (M1) induces a series of descending volleys in the corticospinal tract, which in turn, causes a muscle response referred to as a motor-evoked potential (MEP). Changes in MEP amplitude are thought to reflect adjustments in the physiological strength of corticospinal cell projection onto the spinal motoneuron pool. Further, corticospinal excitability may also be measured by plotting the relationship of the MEP amplitude in response to stimulation at a range of stimulus intensities resulting in a sigmoid recruitment curve (RC). The characteristics of the RC, such as peak slope, stimulus intensity at which the average MEP is midway between minimum and maximum, and the peak height (plateau value), are influenced by the excitability of corticospinal cells underneath the stimulating coil and the spatial distribution of the excitable elements of the M1 and corticospinal pathway [10]. TMS has recently been used in strength training research [11–13]; however, no study has used a paired-pulse TMS protocol to investigate the cortical influence on improved strength development following a period of training with or without WBV. The use of paired-pulse TMS techniques can be used to study intracortical circuitry of the M1. The application of a conditioning stimulus at a set interval activates interneuronal circuits within M1 that influences the excitability of corticospinal neurons to a subthreshold test stimulus delivered up to 20 ms later [14]. The ability of the conditioning stimulus to inhibit (suppress) or facilitate (increase) the motor-evoked potential (MEP) amplitude is dependent on the intensity of the conditioning stimulus (i.e., subthreshold or suprathreshold) and the length of the interstimulus interval (ISI) [15]. Suppression of the MEP with ISIs of 1–5 ms is due to activation of intracortical GABAergic inhibitory interneurons and is termed SICI. Intracortical inhibition (i.e., SICI) has been shown to decrease during voluntary muscle contractions and has been proposed to improve corticospinal drive during intended movement by releasing corticospinal cells from inhibition, improving subsequent excitatory drive to produce the desired movement [16].

Therefore, the purpose of the present study was to utilise a paired-pulse TMS protocol to investigate and compare the effects of 4 wks of strength training with and without WBV on M1 excitability and SICI. It was hypothesized that strength training with superimposed WBV would increase strength and corticospinal excitability and reduce SICI, to a greater extent when compared to strength training alone.

## 2. Methods

### 2.1. Subjects

Twelve healthy individuals, recruited from the university student population (six males and six females) aged between 18 and 27 years, volunteered to participate in the study. Participants, right leg dominant, were systematically and randomly allocated according to gender and baseline strength to either a strength training with superimposed WBV (WBV; *n* = 6,  21 ± 1.6  years) or control group that only performed strength training (*n* = 6,  20 ± 0.8  years). All participants provided written informed consent prior to participation. All procedures were conducted according to the Helsinki Declaration of 1975 granted by the University Human Research Ethics Committee.

### 2.2. Maximum Strength Testing

Maximum voluntary dynamic strength of all participants was determined by a 1RM barbell back squat. All participants completed a warmup that consisted of five minutes moderate aerobic exercise on a cycle ergometer and several warm-up squats with gradually increasing weights. The 1RM test involved performing squats positioned under a power rack (Nautilus Xpload, VA, USA). When performing the squats, the participants were instructed to stand with their feet shoulder width apart, directly under the bar while it rested on their upper back. They were then asked to squat down to a knee angle of 80° controlled using an electromagnetic goniometer (Biometrics, VA, USA), whilst keeping their torso as straight as possible. The load for the first attempt was 5% below the participants expected 1RM. If this lift was successful, it was increased by 2–5% increments until the squat could no longer be successfully lifted, with a period of three minutes rest between attempts. The weight lifted in the last successful attempt was recorded as the 1RM strength. Additionally, maximum voluntary isometric contraction (MVIC) torque of participants was tested during knee extension using an isokinetic dynamometer (Biodex system 4 Pro, Biodex Medical Systems, Shirley, TX, USA). Participants were firmly strapped in, and the test was completed from a passive angle of 60° knee extension. The test consisted of three maximum isometric extension contractions, each lasting for five seconds, with a period of five seconds between each contraction. Strong verbal encouragement was provided to participants, as well as visual feedback of the force exerted, in order to obtain a true maximal effort. The highest peak of the three attempts was recorded as the participants' maximum effort.

### 2.3. Measurement of Anterior Thigh Muscle Thickness

Thickness of the anterior thigh musculature was measured before and after the intervention using a Sonosite ultrasound (Washington, USA). The participants were instructed to lay supine, and a measuring tape was used to measure the distance from the anterior superior iliac spine (ASIS) to the superior border of the patella [17]. A mark was made at the point representing three-fifths of this distance. The 6–15 Hz transducer probe was lubricated with water-soluble transmission gel to minimise underlying soft tissue distortion and then placed on the skin surface perpendicular to the long axis of the thigh on its superior aspect over the mark with minimal pressure. This marking was maintained to ensure accurate data collection. Photos of the initial images were taken to ensure consistency of the image being captured before and after training. The muscle thickness was calculated as the distance (mm) from the femur to the subcutaneous tissue of the anterior thigh musculature, as viewed on a frozen image. It was taken as an average of six consecutive measurements with no more than 5% discrepancy. The testing procedure was found to be reliable, with no significant difference detected between the two testing sessions and a coefficient of variation of less than 1% for the left (*P* = 0.808, *r* = 0.999) and right (*P* = 0.734, *r* = 0.999).

### 2.4. Transcranial Magnetic Stimulation and Electromyography

TMS was applied over the M1 using a BiStim unit attached to two Magstim 200^2^ stimulators (Magstim Co, Dyfed, UK) to produce MEPs in the dominant (right) leg. A standard 90 mm circular coil was held tangential to the skull in an anteroposterior orientation over the vertex of the head so that the left M1 was activated by the counterclockwise current flow. The sites near the estimated centre of the quadriceps femoris (approximately 3-4 cm anterior from the vertex) were explored to find the optimal site at which the largest MEP amplitude was obtained, and this area was marked by “x” in permanent marker. To ensure consistency throughout the study period and reliability of coil placement, the participants maintained the mark before and after testing. For all TMS testing, participants were instructed to exert 10% of their predetermined MVIC, as indicated by a visual line on an oscilloscope representing voluntary knee extension force. Active motor threshold (AMT) was determined as the minimum stimulus intensity required to elicit a MEP in the right rectus femoris of at least 200 *μ*V in 3 out of 5 consecutive trials. AMT was expressed relative to 100% maximum stimulator output (MSO), and the stimulus intensity was altered in 1% increments throughout this process until the appropriate threshold level was achieved.

Surface electromyography (sEMG) activity was recorded from the right rectus femoris muscle using bipolar Ag/AgCl electrodes. These electrodes were placed on the rectus femoris with an interelectrode distance (centre to centre) of 20 mm. The exact area of placement was three fifths of the distance between the ASIS and the upper border of the patella, with the reference (ground) electrode being placed on the patella to ensure no muscle activity was recorded. All cables were fastened with tape to prevent movement artefact. The area of electrode placement was shaven to remove fine hair, rubbed with an abrasive rasp to remove dead skin, and then cleaned with 70% isopropyl alcohol. The exact sites were marked with a permanent marker by tracing around the electrode, and this was maintained for the entire four-week period by both the investigator and participant to ensure consistency of electrode placement relative to the innervation zone. An impedance meter was used to ensure impedance did not exceed 10 kΩ prior to testing. sEMG signals were amplified (×1000) with bandpass filtering between 20 Hz and 500 Hz and digitized at 1.5 kHz for 400 ms, recorded, and analysed using MEGAWIN (Mega Electronics, Finland) software.

### 2.5. Recruitment Curves

Once AMT was established, the stimulus intensities required to establish the TMS recruitment curve (RC) were determined. Each participant was subjected to ten unconditioned stimuli (single-pulse) in a randomised fashion, with the intensity beginning 10% of maximum stimulator output (MSO) below the AMT and increased in 5% of MSO increments until a plateau was observed in MEP amplitude.

### 2.6. Short-Latency Intracortical Inhibition

The protocol for SICI included 15 unconditioned (single-pulse) stimuli elicited at a stimulus intensity 1.2 × AMT, as well as 15 conditioned stimuli to induce SICI. The pair of stimuli to induce SICI consisted of a subthreshold (0.7 × AMT) conditioning stimulus followed by a suprathreshold (1.2 × AMT) test stimulus, with an ISI of 3 ms. SICI was quantified by comparing each of the conditioned (paired-pulse) MEPs to the unconditioned (single-pulse) MEPs at 1.2 × AMT.

### 2.7. M-Waves

Direct muscle responses were obtained from the right rectus femoris by supramaximal percutaneous electrical stimulation of the femoral nerve (approximately 3–5 cm below the inguinal ligament in the femoral triangle) under resting conditions. A Digitimer (Hertfordshire, UK) DS7A constant-current electrical stimulator (pulse duration 1 ms) was used to deliver each electrical pulse. An increase in current strength was applied to the femoral nerve until there was no further increase in the amplitude of sEMG response (M_MAX_). To ensure maximal responses, the current was increased an additional 20% and the average M-wave was obtained from five stimuli delivered at <0.5 Hz.

### 2.8. Strength Training Procedures

Participants allocated to the control group completed a heavy load strength training program involving double leg barbell back squats. The training sessions were performed in a supervised laboratory, three times per week for four weeks (12 sessions in total). The warm-up consisted of five minutes moderate aerobic exercise on a cycle ergometer and several warmup squats with a gradual increase in weight. Participants performed four sets of six to eight repetitions at 80% of 1RM, with three minutes recovery between sets. Existing evidence suggests that this prescription of training variables is ideal to promote maximal strength gains [18]. Each repetition was executed with a three-second eccentric phase immediately followed by a three second concentric phase, which was explicitly controlled through the use of an audible electronic metronome (set at 1 Hz). Participants were verbally encouraged throughout the sessions, and maximal training response was further promoted by employing progressive overload. Once the participants could successfully perform four sets of eight repetitions at the existing weight with correct technique, it was increased by 2–5%.

The participants assigned to the WBV group completed an identical protocol; however, their squats were performed whilst standing on a vertical sinusoidal WBV platform (Power Plate Next Generation, Northbrook, IL, USA). This exposed the participant to a minimum of 36 seconds of vibration per set. The vibration parameters were set at a frequency of 35 Hz and amplitude of 2.5 mm as these characteristics have shown performance improvements in previous studies (for reviews see [1, 2]). All vibration parameters remained unchanged for the duration of the training period, with the principle of progressive overload being applied when appropriate. The vibration amplitude, frequency, and acceleration was set and validated. To validate the vibration characteristics, guidelines from the International Society of Musculoskeletal and Neuronal Interactions were followed [19]. A triaxial accelerometer (Catapult, Melbourne, VIC Australia) was fixed to the edge at the marked foot position, and the peak-to-peak displacement was measured (displacement  = 2.5 mm, acceleration = 32.08 m·s^−2^).

### 2.9. Data Analyses

MEP amplitudes were analysed using MEGAWIN (Mega Electronics, Finland) software after each stimulus was manually flagged with a cursor, providing peak-to-peak values in uV and were then normalised to M_MAX_. In order to determine the peak slope, plateau values, and the stimulus intensity at which MEP amplitude is halfway between top and bottom (*V*_50_), stimulus intensity was plotted against MEP amplitude (% of M_MAX_) for all participants to create RC and then fitted with a nonlinear Boltzmann sigmoidal equation using Prism5 (GraphPad Software Inc., CA, USA):

(1) $$\mathrm{MEP}\left(s\right)=\text{Bottom}+\frac{\left(\text{Top}-\text{Bottom}\right)}{1+\exp\left(\left(V_{50}-x\right)/\text{Slope}\right)},\;$$

where *s* represents stimulus intensity, Top represents the MEP plateau value (peak height), *V*_50_ represents the stimulus intensity at which MEP amplitude is halfway between top and bottom, Slope represents the steepness of the curve.

SICI was quantified by dividing the average paired-pulse MEP by the average single-pulse MEP at 1.2 × AMT and multiplying by 100.

### 2.10. Statistical Analyses

All data were screened for normality using the Shapiro-Wilk and the Kolmogorov-Smirnov tests, and were found to be normally distributed. Consequently, two-way (group × time) repeated-measures ANOVAs were used to determine any significant differences between and within groups for each dependant variable (1RM squat strength, SICI, stimulus output required to evoke AMT, MEP amplitude at 1.2 × AMT [% of M_MAX_], *MEP*⁡_MAX_  [% of M_MAX_], and properties of the RC). If the ANOVA indicated significant differences or interactions, post-hoc comparisons were completed using Bonferroni's correction (*P* ≤ 0.016). Means and standard error (SE) were calculated for all dependant variables. Intraclass correlation coefficients (ICCs), CoV, and paired *t*-tests were used to determine the reliability of the ultrasound testing protocol. The level of significance for tests was set at *P* ≤ 0.05.

## 3. Results

### 3.1. Muscle Thickness

The means (±SE) for muscle thickness of the anterior thigh musculature are displayed in Table 1. There were no differences in anterior thigh muscle thickness detected between the groups at baseline (left leg: *F*_1,10_ = 0.006; *P* = 0.939; right leg: *F*_1,10_ = 0.342; *P* = 0.572). There was no main effect for time (left leg: *F*_1,10_ = 1.148; *P* = 0.309; right leg: *F*_1,10_ = 2.837; *P* = 0.123), group (left leg: *F*_1,10_ = 4.205; *P* = 0.067; right leg: *F*_1,10_ = 3.686; *P* = 0.084), or group by time interaction for anterior thigh muscle thickness following the intervention (left leg: *F*_1,10_ = 0.001; *P* = 0.980; right leg: *F*_1,10_ = 2.837; *P* = 0.123).

### 3.2. Maximal Voluntary Isometric Contraction (MVIC)

Table 1 presents the mean (±SE) for MVIC before and after intervention. There were no differences in MVIC between groups at baseline (*F*_1,10_ = 0.050; *P* = 0.827). There was also no main effect for time (*F*_1,10_ = 2.962; *P* = 0.116), group (*F*_1,10_ = 0.674; *P* = 0.431), or group by time interaction (*F*_1,10_ = 0.514; *P* = 0.490) detected.

### 3.3. Dynamic Strength (1RM)

**Figure 1:**
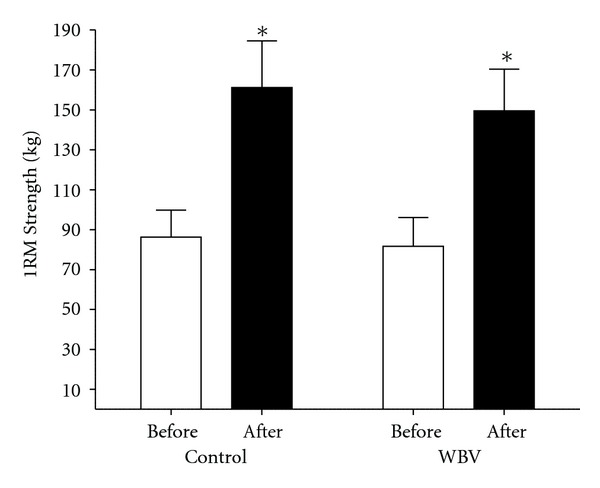
Mean (±SE) absolute change in dynamic single repetition maximum (1RM) squat strength (kg) before and after the 4 wk intervention. The training protocol resulted in 1RM increases in both groups (*P* < 0.05), as denoted by an asterisk. However, the magnitude of change between training groups was not significantly different (*P* = 0.753).

There was an 86.96% and 83.16% increase in 1RM strength in the control and WBV groups, respectively, following the intervention (Figure 1). Groups did not differ in 1RM strength at baseline (*F*_1,10_ = 0.017; *P* = 0.899). There was a main effect for time (*F*_1,10_ = 98.550; *P* < 0.001), however, no main effect for group (*F*_1,10_ = 0.104; *P* = 0.753) or group by time interaction detected following the intervention (*F*_1,10_ = 75.260; *P* = 0.633).

### 3.4. Active Motor Threshold and Motor-Evoked Potentials

Mean (±SE) TMS stimulus output required to evoke AMT for the right rectus femoris was 44.7 ± 2.0% and 46.33 ± 3.4% for control and WBV, respectively, prior to the intervention, and 41.0 ± 2.3% and 44.5 ± 3.3% following the intervention. There were no differences in TMS stimulus output required to evoke AMT between groups at baseline (*F*_1,10_ = 1.416; *P* = 0.262). There was a main effect for time (*F*_1,10_ = 6.764; *P* = 0.026), however, no main effect for group (*F*_1,10_ = 0.458; *P* = 0.514) or group by time interaction (*F*_1,10_ = 0.752; *P* = 0.406) following the intervention.

**Figure 2:**
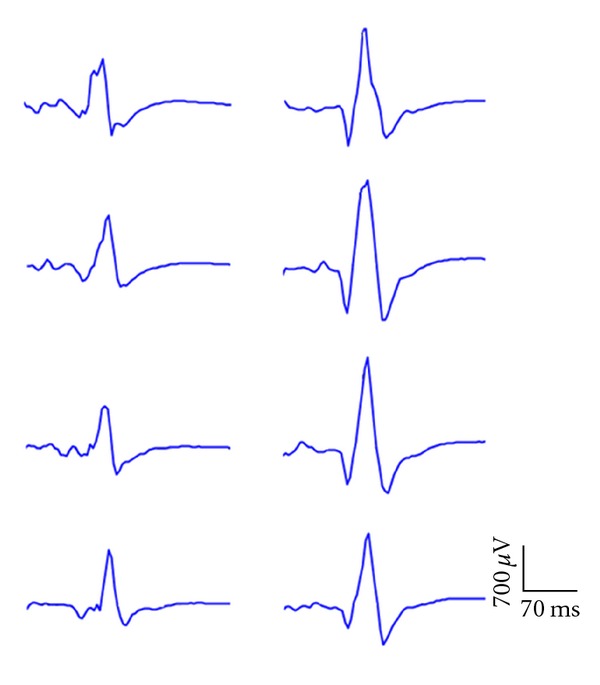
MEPs obtained from the right rectus femoris of one participant in the WBV group obtained at 1.2 × AMT. The four sweeps on the left show preintervention MEPs, and the four sweeps on the right show facilitated MEPs following 4 wks of training. These results were similar for both groups.

Mean (±SE) absolute values for MEP amplitude at 1.2 × AMT normalised to the M-wave (M_MAX_) for the right rectus femoris for all groups before and after intervention are shown in Table 2. There were no differences in MEP amplitude at 1.2 × AMT (% of M_MAX_) between groups at baseline (*F*_1,10_ = 0.114; *P* = 0.742). There was a main effect for time (*F*_1,10_ = 22.462; *P* = 0.001), however, no main effect for group (*F*_1,10_ = 0.000; *P* = 0.985) or group by time interaction (*F*_1,10_ = 0.601; *P* = 0.456) following the intervention. The raw MEPs obtained from the right rectus femoris of one participant in the WBV group obtained at 1.2 × AMT are shown in Figure 2.

Mean (±SE) absolute values and percentage change for *MEP*⁡_MAX_ (% of M_MAX_) for both groups before and after intervention are shown in Table 2. There were no differences in *MEP*⁡_MAX_ normalised to M_MAX_ between groups at baseline (*F*_1,10_ = 1.021; *P* = 0.336). There was a main effect for time (*F*_1,10_ = 71.876; *P* < 0.001), however, no main effect for group (*F*_1,10_ = 1.161; *P* = 0.696) or group by time interaction (*F*_1,10_ = 0.309; *P* = 0.590) following the intervention.

RCs were constructed for each participant to determine values for the estimated peak slope of the curve, *V*_50_, and the plateau values of the curve (peak height). There were no differences in estimated peak slope between groups at baseline (*F*_1,10_ = 0.143; *P* = 0.714). Further, there were no main effects for time or group (*F*_1,10_ = 0.421; *P* = 0.531 and *F*_1,10_ = 1.917; *P* = 0.196, resp.) and no group by time interaction following the intervention (*F*_1,10_ = 0.570; *P* = 0.468).

No differences in *V*_50_ were detected between the groups at baseline (*F*_1,10_ = 0.000; *P* = 0.988). There was a main effect detected for time (*F*_1,10_ = 12.256; *P* = 0.006), but no main effect for group (*F*_1,10_ = 0.040; *P* = 0.845) or group by time interaction (*F*_1,10_ = 0.082; *P* = 0.781) detected for *V*_50_ following the intervention.

**Figure 3:**
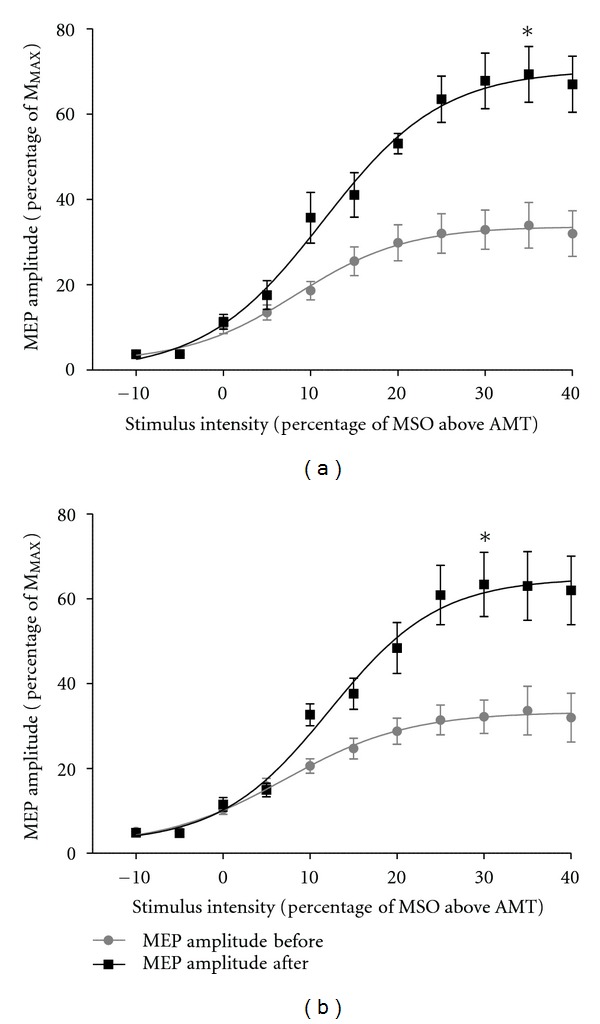
Mean (±SE) MEP (% of M_MAX_) recruitment curves for the right rectus femoris for the control group (a) and WBV group (b), respectively. The grey curve represents MEP data collected during pretesting, and the black curve represents data collected during posttesting. AMT is represented as *x* = 0, with data taken at stimulus intensities 10% of maximum stimulator output (MSO) below AMT (*x* = −10), increasing in increments of 5% of MSO until MEP amplitude (% of M_MAX_) reached a plateau (peak height). There were no differences in *V*_50_ (*P* = 0.988) or peak height (*P* = 0.419) of the curve between groups at baseline. However, there were 64.77% and 73.93% increases in *V*_50_ (*P* = 0.006) and 105.16% and 112.22% increases in peak height (*P* < 0.001) following the intervention, as denoted by an asterisk. There were no significant differences between groups for either *V*_50_ (*P* = 0.845) or peak height (*P* = 0.888) following the intervention.

The plateau value (peak height) of the curve was also compared before and after intervention, with no differences detected between groups at baseline (*F*_1,10_ = 0.710; *P* = 0.419). There was a main effect for time (*F*_1,10_ = 68.820; *P* < 0.001); however, there were no main effects for group (*F*_1,10_ = 0.021; *P* = 0.888) or group by time interaction (*F*_1,10_ = 0.078; *P* = 0.786) (Figure 3).

### 3.5. Short-Latency Intracortical Inhibition

**Figure 4:**
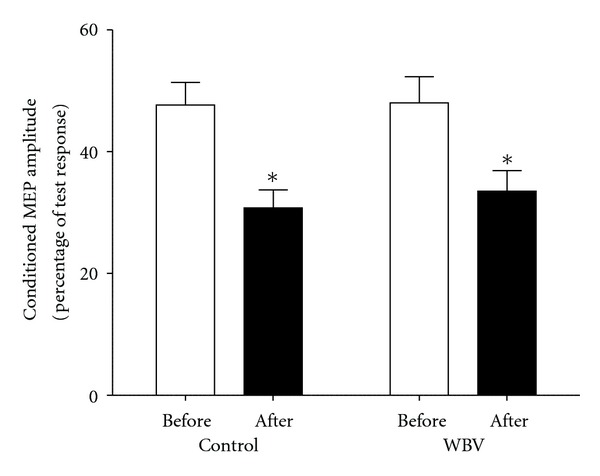
Mean (±SE) absolute change in SICI expressed as the ratio between conditioned MEPs and test MEPs for both groups before and after the 4 wk intervention. The training protocol resulted in significant reductions in SICI for both training groups (*P* < 0.05). However, the magnitude of change between training groups was not significantly different (*P* = 0.727).

Table 2 and Figure 4 contain mean (±SE) absolute values for SICI (as a ratio between conditioned MEP amplitudes and test MEP amplitudes) for both groups, before and after intervention. There were no differences in SICI between groups at baseline (*F*_1,10_ = 0.217; *P* = 0.651). There was a main effect for time (*F*_1,10_ = 33.171; *P* < 0.001), however, no main effect for group (*F*_1,10_ = 0.129; *P* = 0.727) or group by time interaction detected following the intervention (*F*_1,10_ = 0.193; *P* = 0.670).

## 4. Discussion

The purpose of the present study was to quantify the strength improvements and corticospinal adaptations confined to the M1 following 4 wks of identical heavy load strength training either with or without superimposed WBV. There were several important findings. Foremost, this is the first study to assess the effectiveness of superimposing traditional strength training with WBV to elicit superior strength development and neural adaptations. Both groups experienced a significant increase in 1RM strength and corticospinal excitability, as well as a significant reduction in SICI with no corresponding change in muscle thickness following the 4 wk intervention. However, the novel finding of the current study was that 4 wks of WBV training did not offer any appreciable advantage in any outcome measure when compared to identical strength training without superimposed vibration.

### 4.1. Dynamic Strength (1RM)

WBV training did not lead to greater increases in strength development compared to the control group. Whilst it has been shown that exposure to vibration can lead to enhanced strength development in both the upper and lower body [3–6], there are a number of possible explanations as to why this expected result did not occur in the current study. It has been shown that the true frequency or amplitude of WBV imposed on the body can differ from the preset values of the vibration device, particularly when using additional loads/weights [20]. Whilst this was not directly quantified in the current study, Pel et al. [20] reported a 10-fold reduction in transmission of WBV-induced acceleration from the ankle to the knee/hip at a range of frequencies above 20 Hz. Additionally, a recent study investigating the effects of WBV and conventional loaded squat exercises on muscle activation showed no differences in sEMG at various sites of the body, across a number of training loads and vibratory accelerations [21]. Furthermore, it has been speculated that high muscle activity levels associated with muscle stiffness is a possible explanation for modulation of strength development following WBV exposure [20]. Although muscle stiffness was not measured in the current study, the heavy training load utilised (i.e., 80% of maximum voluntary strength) is likely to have promoted increased muscle activity and muscle stiffness which acted to reduce soft tissue resonance and consequently dampened the vibratory effects imparted upon the trained muscles [22]. Given the identical prescription of training load for both groups, it appears that the mechanism for increased strength in the current study was facilitated by the heavy training load and not the exposure to superimposed vibration. Therefore, the present findings show that superimposed vibration confers no additional benefit compared to strength training alone on modulating strength development.

### 4.2. Corticospinal Excitability

Following 4 wks of heavy load strength training, there were significant changes in the strength of corticospinal projection (i.e., corticospinal excitability) as demonstrated by increases in MEP amplitude at 1.2 × AMT, *MEP*⁡_MAX_, *V*_50_, and peak height of the RC. However, the main finding of the current study was that 4 wks heavy-load strength training resulted in similar increases in both training groups, showing that WBV training did not preferentially modulate corticospinal excitability. This further suggests that the neural mechanisms modulating improved strength following a period of heavy-load strength training were similar for both groups.

Whilst there is evidence of increased corticospinal excitability from direct vibration [23, 24], only one study has investigated the effect of acute WBV on corticospinal excitability and found an increase in MEP amplitude [25]. Therefore, the current study is the first to investigate the training-related effects of WBV on corticospinal and M1 excitability. Given that there were no significant differences between the control and WBV groups and the only difference in the training interventions was the addition of WBV, the physiological responses observed must be attributed to factors beyond WBV exposure. Increased strength following short-term strength training is thought to occur as a result of training-induced changes in the neural control of the trained muscles [11, 26]. It is well accepted that these changes in neural activity are dependent on the attention required to perform tasks of increasing difficulty [27–29]. This has been supported in TMS studies that have shown changes in synaptic activity following a period of motor skill training [27, 29], with more demanding tasks leading to greater activation and higher facilitation than less demanding tasks [30, 31]. For example, Perez et al. [27] showed that motor skill training elicited an increase in corticospinal and M1 excitability with no comparable changes in the same task performed in a nonskilled manner. Skill training is defined as the acquisition and subsequent refinement of novel combinations of movement sequences [32]. Based on this rationale, it has been suggested that some strength training programs may be considered a form of skill training [11, 33]. In the present study, all participants were untrained and performed unfamiliar multijoint strength training with explicitly controlled repetition timing and depth. These factors added an element of skill acquisition to their strength training programs and most likely support a task-dependant response in corticospinal and M1 excitability.

### 4.3. Short-Latency Intracortical Inhibition

Cortical interneurons elicit either an inhibitory or excitatory influence on output from the M1, and changes in their behaviour are highly dependent on the nature of training or experience [34, 35]. It is known that, when SICI is reduced, the balance between excitation and inhibition within intracortical circuits facilitates motor output [16]. This study provides the first line of evidence that this occurs following short-term, heavy-load strength training with and without superimposed WBV. Furthermore, the comparable reduction in intracortical inhibition between the control and WBV group demonstrates that the mechanisms modulating strength development following a period of controlled heavy-load strength training are not altered through exposure to WBV.

The cortical projections to trained muscles are likely to be suppressed under normal conditions due to inhibitory mechanisms, but training, particularly involving skilled practice, induces a reduction in this inhibition, thus, strengthening connections between interneurons and corticospinal neurons [35, 36]. Pascual-Leone et al. [35] was the first to demonstrate that TMS responses are altered due to practice and suggested that long-term potentiation effects and reductions in intracortical inhibition result in strengthening of existing neural connections within the M1. This line of evidence supports the current findings by improved voluntary motor drive to the trained muscles. In addition, we have demonstrated that, following strength training with and without WBV, the MEPs evoked by paired-pulse TMS testing were significantly facilitated (reduced SICI), demonstrating increased M1 excitability. The potential mechanisms for this following training include synaptogenesis or unmasking of silent synapses (disinhibition), confined to the M1, and improved strength of existing corticospinal connections due to a reduction in inhibition (for review see [37]). The facilitated test response provides evidence of synaptic plasticity at a cortical level, with membrane potentials becoming closer in proximity to their firing thresholds, as well as improved synaptic efficacy between these neurons and the axons activated by TMS [38]. Such plasticity has been observed in studies using direct vibration [39, 40] and prior to and during voluntary muscle contractions [41, 42]. The findings of this study are unique as they are the first to show the training-related effects of reduced SICI and increased M1 excitability as a result of task-dependant changes associated with heavy-load strength training. Importantly, the present finding suggests that superimposed WBV does not further reduce intracortical inhibition or increase M1 excitability beyond that of conventional strength training. Taken together, this data demonstrates that the changes in cortical plasticity are due to the effects of strength training and not the application of vibration.

## 5. Conclusions

This study demonstrated an increase in 1RM strength, increased corticospinal excitability, and a reduction in inhibition confined to the M1 in both training groups. This provides evidence that the characteristics of the training itself (heavy loads, unfamiliarity, complexity) appear to be fundamentally important in maximising cortical plasticity from training and that WBV is ineffective as a stimulus to facilitate these adaptations when compared to heavy-load strength training. Additionally, further investigation into the use of WBV as a training modality is required. This potentially includes investigations into the most appropriate prescription of WBV, including frequency and amplitude (perhaps individualised to each participant), the interaction of various training loads on the vibratory stimulus, and its effect on neuromuscular function. This study utilised a short-term heavy-load strength training protocol, which potentially dampened the constant vibratory stimulus through increased muscle activity associated with the high-intensity load itself. Therefore, studies investigating the neural adaptations to low-moderate intensity strength training are required, as this has implications for the prescription of training to populations such as the elderly or otherwise impaired.

## Figures and Tables

**Table 1:** Mean ± SE values and percentage change for maximum voluntary isometric contraction torque (N·m) and muscle thickness of the left and right anterior thigh.

Group	MVIC peak torque (N·m)			Left muscle thickness (mm)			Right muscle thickness (mm)		
	Before	After	Change (%)	Before	After	Change (%)	Before	After	Change (%)
Control	205.67 ± 18.59	215.00 ± 20.06	**4.54**	41.14 ± 2.06	41.20 ± 2.14	**0.14**	40.48 ± 2.35	40.60 ± 2.36	**0.30**
WBV	176.33 ± 16.75	199.00 ± 25.87	**12.85**	34.90 ± 2.21	34.96 ± 2.20	**0.17**	34.41 ± 2.16	34.41 ± 2.14	**0.01**

**Table 2:** Mean ± SE values and percentage change for corticospinal responses: MEP amplitude at 1.2 × AMT (% of M_MAX_), MEP_MAX_ (% of M_MAX_), and conditioned MEP amplitude as a percentage of the test response (short-latency intracortical inhibition (SICI)).

	MEP amplitude at 1.2 × AMT			MEP_MAX_			SICI		
Group	(% of M_MAX_)			(% of M_MAX_)			(% of test response)		
	Before	After	Change (%)	Before	After	Change (%)	Before	After	Change (%)
Control	16.32 ± 1.61	35.29 ± 6.85	**116.24**	33.45 ± 4.80	70.96 ± 6.73	**112.10**	47.67 ± 3.72	30.77 ± 2.96	**−35.45**
WBV	18.91 ± 1.38	32.55 ± 2.52	**72.14**	32.83 ± 3.71	65.72 ± 7.68	**100.17**	48.02 ± 4.29	33.52 ± 3.37	**−30.20**
